# Factors influencing use of contraceptives among literate married women in Ogbomoso South Local Government Area, Oyo State

**DOI:** 10.4314/ahs.v24i1.15

**Published:** 2024-03

**Authors:** Lateef Omotosho Adegboyega, Adeola Abideen Adewusi

**Affiliations:** 1 Department of Counsellor Education, Faculty of Education, University of Ilorin, Ilorin, Nigeria; 2 National Commission for Museums and Monuments, Ilorin, Kwara State, Nigeria

**Keywords:** Factors, Use of Contraceptives, Literate married women

## Abstract

**Background:**

Contraceptives uses are issues of concern around the world due to the adverse effects of unsafe sexual behaviours, such as unwanted pregnancies and sexually transmitted diseases among women.

**Objective:**

To investigate the factors influencing use of contraceptives among literate married women in Ogbomoso South Local Government Area, Oyo State. The study also examined whether the variables of age, religion and educational qualification would influence the respondent's view

**Methods:**

Descriptive survey design was adopted for the study. Purposive sampling technique was adopted to draw a total of 210 respondents. A questionnaire was used to collect data for the study. Mean and rank order was used to answer the research question while Analysis of Variance was used to test the hypotheses at 0.05 level of significance.

**Results:**

Findings revealed that factors influencing contraceptive use among literate married women are educational qualification, health condition and number of children among others. Findings also revealed that there were no significant differences in the factors influencing use of contraceptives among literate married women based on age and religious affiliation while significant difference was found in educational qualification.

**Conclusion:**

Majority of the respondents attested to the factors influencing contraceptive use among literate married women. Based on the findings of the study, it was recommended that contextual and cultural considerations are recommended for comprehensive understanding of factors influencing contraceptive use among Nigerian women, educative interventions by service providers on the necessity of continuous contraception even at older age before menopause should be recommended.

## Introduction

Contraceptives uses are issues of concern around the world due to the adverse effects of unsafe sexual behaviours, such as unwanted pregnancies and sexually transmitted diseases among women. Contraceptive by definition is the use of device or drugs to prevent conception in sexual intercourse^1^. Contraceptive methods are the techniques used in preventing unwanted pregnancy and other STIs (Sexually Transmitted Infections). There are varieties of birth control methods available to choose from, although most options are for women. However, selecting methods is a personal decision that involves consideration of many factors, including convenience, reliability, accessibility, cost, side effects and reversibility (whether the method is temporary or permanent). For instance, some people may prefer a birth control option that provides continuous protections against pregnancy, while other may prefer a method that only prevents pregnancy during a single act of sexual intercourse^2^.

Birth control methods work in different ways to prevent pregnancy. Some methods prevent sperm from meeting eggs; some affect a woman's hormones, altering her reproductive circles, while other birth control methods involve behaviours that alter sexual activity in ways that lessen the chance for pregnancy^3^. Some of them are barriers methods like the male condom, female condom (vaginal pouch), diaphragm, cervical cap and spermicides. There is also the intrauterine device (IUD), hormonal contraceptives like birth control pill, hormonal implants, contraceptives injections, contraceptives ring and contraceptives patch.

Condom is the most commonly used contraceptive among students (boys and girls) in Nigeria. Condom use is estimated to be 43 percent and use of rhythm, 31 percent. Thus, the use of condoms and vaginal spermicides continue to be recommended for all sexually active students to decrease the risk of contracting sexually transmitted diseases. In addition to the latex condom and other barrier contraceptives, the following like diaphragm, cervical cap, vaginal sponge; female condom and vaginal spermicides are recommended for most students^4^. In Nigeria, unintended intercourse is the primary cause of unwanted pregnancies, and many students with unwanted pregnancies decide to end them by abortion^5^,^6^. Since abortion is illegal in Nigeria (except on the grounds to save the life of the mother), as a result, many abortions are done in unsafe environments and these illegal abortions have severe consequences that can be life threatening, sometimes, leading to maternal deaths^7^,^8^,^9^.

Unexpected or unplanned pregnancy poses a major public health challenge on women of reproductive age, especially in developing countries. It has been estimated that of the 210 million pregnancies that occur annually worldwide, about 80 million (38%) are unplanned, and 46 million (22%) ends in abortion. Many factors contribute to unwanted pregnancy in Nigeria, and a very common factor is the low level of contraceptive use. Among Nigerian women of reproductive age, one in seven (14%) have tried to have an abortion, and one in ten (10%) have actually ended an unwanted pregnancy, suggesting up to 760,000 induced abortions annually^10^.

The use of modern contraceptive methods translates into the prevention of unwanted pregnancy and subsequent abortions. If contraceptive use in the population increases among Nigerian men and women who are sexually active, there will be a significant reduction in unwanted pregnancies and abortions leading to reduced maternal mortality. Research carried out in Nigeria by National Population Commission (2004) indicated that more than 60 percent of students with an unplanned pregnancy are not using any form of contraception.

Contraceptive prevalence rates have correlated with maternal mortality and it has been shown that countries with low contraceptive prevalence rates are also countries with very high maternal mortality ratios^11^. Nigeria has one of the highest maternal mortality ratios in sub-Saharan Africa, and ranks as the country with the second highest number of maternal deaths in the world^12^. However, illegal and unsafe abortions contribute 20-40 percent of about 60,000 maternal deaths that occur yearly in Nigeria^4^. Considering the foregoing, the researchers deem it is very necessary and important that empirical study be conducted on factors influencing use of contraceptives among literate married women in Ogbomoso South Local Government Area, Oyo State.

### Statement of the problem

General awareness of family planning is almost universal, at 95 percent of women of reproductive age. Yet the level of contraception remains low at less than half of all currently married women using any method. A substantial proportion of married women still have an unmet need for family planning, which was at 25.6 percent in 2008.

Physical and physiological developments are demanding and challenging tasks for individual. Centre for Disease Control and Prevention 2010 discovered that only thirty-nine percent of all sexually active students did not use any form of contraceptive at last sexual intercourse. Moreover, only few of those who used contraceptives possessed little (or no information) about the reproduction anatomy. Various researches have shown that friends, schoolmates and the media are the common sources of information about sexual and reproductive matters, while parents, guardian and schools are the least common source.

Unwanted pregnancy and abortion constitute the major challenges arising from an unwanted and unplanned sexual intercourse. Eruesegbefe observed that the news of unexpected pregnancy is, in most cases, followed by the trauma of broken promises, emotional trauma, interrupted educational pursuits, shattered relationships and unfulfilled potentials. Eruesegbefe also reported further that early motherhood, as consequence of an adolescent student being pregnant, limits educational and career opportunities and perpetuates the inequality that already exists between the sexes.

Thus, sporadic premarital sexual intercourse and poor use or non-use of contraceptives has perhaps resulted into unwanted pregnancy, illegal abortion and child abandonment. According to Bankole, Oye-Adeniran and Singh, hundreds of thousands of abortions are performed in Nigeria each year by doctors and nurses working mostly in private hospitals or clinics; and many others are performed by untrained practitioners or women themselves under risky conditions. Ogunsanya also reported that “girls under 20 years old suffer pregnancy and delivery complications such as anaemia, premature delivery, prolonged labour and Vesico Vaginal Fistula (VVF) than students aged 20 or more.

Aliyu and Mburza examined sexual behaviour and contraceptive use among student nurses, school of nursing, university of Maiduguri. The results revealed that 21 (17 percent) female and 15 (12.3 percent) male student nurses were involved in sexual intercourse before marriage. The results also revealed further that 13 (10.7 percent) of females and 10 (8.2 percent) of the male student nurses used contraceptives. The female student nurses constituted the majority, 73 (59.3 percent) and only 26 (21.3 percent) of the males reported they have never used contraceptives. To the best of researcher's knowledge, there are only few studies that have been carried out in Ogbomoso on contraceptive use among literate married women. In view of this, this study investigates factors influencing use of contraceptives among literate married women in Ogbomoso South Local Government Area, Oyo State.

### Research question

- What are the factors influencing use of contraceptives among literate married women in Ogbomoso South Local Government Area, Oyo State?

### Research hypotheses

The following hypotheses are postulated to guide the conduct of the study:

1. There is no significant difference in the factors influencing use of contraceptives among literate married women in Ogbomoso South Local Government Area, Oyo State based on age.
2. There is no significant difference in the factors influencing use of contraceptives among literate married women in Ogbomoso South Local Government Area, Oyo State based on religion.
3. There is no significant difference in the factors influencing use of contraceptives among literate married women in Ogbomoso South Local Government Area, Oyo State based on educational qualification.

### Research design

The research design that was adopted for this study is the descriptive survey method. This method helps in collection of data for the purpose of describing and interpreting existing conditions, prevailing practices, attitudes and ongoing process. The study was conducted between February, 2022 and November, 2022.

### Population, sample and sampling procedure

Population is the entire group which the researcher is interested in gaining information from and upon which subsequent conclusions are drawn^15^. Therefore, the population of this study covered all literate married women in Ogbomoso while the target population for the study were the selected literate married women in Ogbomoso North Local Government Area, Oyo State. A total of 210 literate married women in Ogbomoso were chosen using random sampling technique.

### Instrumentation

A questionnaire titled “Factors Influencing Contraceptive Use Questionnaire” (FICUQ) was used in obtaining data. The Questionnaire consists of two sections (A and B). Section A entails demographic data of the respondents such as age, religion and educational qualification. Section B contained 20 items on the respondents' factors influencing contraceptive use; the section was patterned after a Four Point Likert-type Rating Scale of: SA- Strongly Agree, A- Agree, D- Disagree and SD- Strongly Disagree. The respondents were expected to rate the items as applicable in the section.

### Psychometric properties of the instrument Validity

Validity of any instrument is the extent to which an instrument measures what it purports to measure. Therefore, in order to ascertain the validity of the instrument, the researchers gave the questionnaire to health practitioners and counsellors for vetting. The corrections made were dully observed and effected and the instrument was confirmed as being valid for the study.

### Reliability

Reliability means the extent to which the results obtained from the test can be relied upon as the true score^15^. In order to ascertain the reliability of the instrument (FICUQ), the test re-test method was used to ascertain the reliability of the instrument. A total of forty (40) questionnaire forms were administered at an interval of four-weeks, another forty (40) questionnaire forms were re-administered to the same group of respondents; the two sets of scores obtained from the two administrations were correlated by Pearson's Product Moment Correlation which yielded 0.85 to make the instrument reliable for use.

### Ethical issues

The researchers sought the permission of the Ethical committee of the University of Ilorin in which approval (dated 22^nd^ of November, 2021) was given by the committee to conduct the study with the approval number UERC/ASN/2021/389. The objective of the research was clearly explained to the respondents by attaching informed consent forms to the questionnaire. Respondents were assured of utmost anonymity and confidentiality of any information provided on the questionnaire form. Each respondent's identity was protected as their names were not requested.

### Method of data analysis

The data generated were analysed by percentage for socio-demographic characteristics (see Table 1). Mean and rank order were used to analysed the research question (see Table 2). Hypotheses 1, 2 and 3 were tested with Analysis of Variance (ANOVA). ANOVA is an inferential statistical measure for comparing the means of more than two groups^15^. Therefore, all hypotheses in the study were tested at 0.05 level of significance.

**Table 1:** Percentage distribution of respondents based on age, religion and educational qualification

Age	Frequency	Percentage
20-30 years	150	71.4
31-40 years	36	17.1
41 years and above	24	11.4
Total	210	100


Religion	Frequency	Percentage
ATR	6	2.9
Christianity	61	29.0
Islam	143	68.1
Total	210	100


Qualification	Frequency	Percentage
SSCE	12	5.7
NCE/OND	34	16.2
HND/Degree	128	61.0
Postgraduate	36	17.1
Total	210	100

**Table 2:** Mean and rank order on factors influencing use of contraceptives

Item No.	Factors influencing contraceptive use are:	Mean	Rank
2	educational qualification	3.77	1^st^
10	health condition	3.69	2^nd^
5	number of children	3.46	3^rd^
8	access to contraceptives	3.41	4^th^
19	sexual interest	3.40	5^th^
18	nature of work	3.39	6^th^
15	societal norms	3.39	6^th^
6	spouse interest	3.33	8^th^
20	hormone influence	3.23	_9_th
12	financial reasons	3.16	10^th^
3	age	3.14	10^th^
14	women empowerment	3.11	12^th^
16	influence of family member	3.06	13^th^
11	child spacing method	3.06	13^th^
4	Environment	3.00	15^th^
9	awareness on contraceptive uses	2.94	16^th^
13	fertility norms	2.94	16^th^
17	family planning programmes	2.83	18^th^
1	Religion	2.68	19^th^
7	socio cultural factors	2.51	20^th^

## Results

### Demographic data

This section presents the results of data obtained on the respondents in frequency counts and percentages.

Table 1 shows the distribution of respondents' age range, religious affiliation and educational qualification. The table reveals that 71.4% (150) of the respondents were between 18-35 years old, 17.1% (36) of the respondents were between 36-45 years old while 11.4% (24) of the respondents were 46 years and above. This also indicates that respondents who were between the ages 18-35 years old participated more than other age groups.

The table also shows that 2.9% (6) of the respondents were practicing African Traditional Religion, 29.0% (61) of the respondents were practicing Christianity while 68.1% (143) of the respondents were practicing Islam. This indicates that respondents practicing Islam participated more in the study.

Also, the table shows the distribution of respondents' educational qualification. The table reveals that 5.7% (12) of the respondents had SSCE, 16.2% (34) of the respondents had NCE/OND, 61.0% (128) of the respondents had HND/Degree while 17.1% (36) of the respondents had postgraduate degree. This also indicates that respondents who had HND and 1st degree participated more in this study.

### Research question

What are the factors influencing use of contraceptives among literate married women in Ogbomoso South Local Government Area, Oyo State?

Table 2 presents the mean and rank order of the factors influencing contraceptive use among literate married women. The Table indicates that Items 2, 10 and 5 with mean scores of 3.77, 3.69 and 3.46 states those factors influencing contraceptive use are “educational qualification, health condition and number of children respectively. While items 17, 1 and 7 with mean scores of 2.83, 2.68 and 2.51 states those factors influencing contraceptive use are “family planning programmes, Religion and socio-cultural factors respectively. Since all of the twenty items have mean scores that are above the mi-mean score of 2.50, then it can be said that respondents attested to factors influencing contraceptive use among literate married women listed on the table.

### Hypotheses testing

Three null hypotheses were postulated and tested for this study. The hypotheses were tested using ANOVA statistical method at 0.05 level of significance.

**Hypothesis one:** There is no significant difference in the factors influencing use of contraceptives among literate married women in Ogbomoso South Local Government Area, Oyo State based on age.

Table 3 shows that the calculated F-value of 1.42 is less than the critical F-value of 3.00 with the corresponding p-value of .318 which is greater than 0.05 level of significance. The null hypothesis is not rejected since the p-value is greater than the alpha level. Hence, there is no significant difference in the factors influencing use of contraceptives among literate married women in Ogbomoso South Local Government Area, Oyo State based on age.

**Table 3:** Analysis of variance (ANOVA) showing the respondents' perception of the factors influencing use of contraceptives among literate married women based on age

Source	df	SS	Mean Squares	Cal. F-value	Crit. F-value	p-value
Between Groups	2	438.620	219.310	1.42	3.00	.318
Within Groups	207	11277.837	154.482			
Total	209	11716.457				

**Hypothesis two:** There is no significant difference in the factors influencing use of contraceptives among literate married women in Ogbomoso South Local Government Area, Oyo State based on religion.

Table 4 shows that the calculated t-value of 1.18 is less than the critical t-value of 3.00 with the corresponding p-value of .307 which is greater than 0.05 level of significance. The null hypothesis is not rejected since the p-value is greater than the alpha level, therefore, there is no significant difference in the factors influencing use of contraceptives among literate married women in Ogbomoso South Local Government Area, Oyo State based on religion.

**Table 4:** Analysis of variance (ANOVA) showing the respondents' perception of the factors influencing use of contraceptives among literate married women based on religion

Source	df	SS	Mean Squares	Cal. F-value	Crit. F-value	p-value
Between Groups	2	133.061	66.530	1.18	3.00	.307
Within Groups	207	11583.396	55.958			
Total	209	11716.457				

**Hypothesis three:** There is no significant difference in the factors influencing use of contraceptives among literate married women in Ogbomoso South Local Government Area, Oyo State educational qualification.

Table 5 shows that the calculated t-value of 36.33 is greater than the critical t-value of 2.60 with the corresponding p-value of .000 which is less than 0.05 alpha level of significance. The null hypothesis is rejected since the p-value is less than the alpha level. Hence there is significant difference in the factors influencing use of contraceptives among literate married women in Ogbomoso South Local Government Area, Oyo State based on educational qualification.

**Table 5:** Analysis of variance (ANOVA) showing the respondents' perception of the factors influencing use of contraceptives among literate married women based on educational qualification

Source	df	SS	Mean Squares	Cal. F-value	Crit. F-value	p-value
Between Groups	3	4054.401	1351.467	36.33*	2.60	.000
Within Groups	206	7662.056	37.194			
Total	209	11716.457				

In order to determine the mean value(s) that caused the significant difference observed in the ANOVA results of Table 5, the Duncan Multiple Range Test (DMRT) was used as a post-hoc test. The results of the DMRT procedure are displayed in Table 6.

**Table 6:** Duncan multiple range test (DMRT) showing the differences of the respondents' perception of the factors influencing use of contraceptives among literate married women based on educational qualification

Qualification	N	Mean	Group	Duncan groupings
Postgraduate	36	69.39	1	A
SSCE	12	66.33	2	B
HND/Degree	128	63.96	3	C
NCE/OND	34	54.62	4	D

Table 6 showed the DMRT indicating the significant difference noted in the ANOVA on Table 5. Group 1 with a mean score of 69.39 significantly differed from Group 2 with a mean score of 66.33, but significantly differed from Group 3 with a mean score of 63.96. but also significantly differed from Group 4 with a mean score of 54.62. All the groups differed from one another but the significant difference noted was as a result of the mean of Group 1 with the highest mean score, hence the significant difference noted in the ANOVA on Table 5 was brought about by respondents who had postgraduate degree therefore, the hypothesis is rejected.

## Discussion

The finding of the study revealed that factors influencing contraceptive use among literate married women are educational qualification, health condition and number of children among others. This finding corroborated the finding of Adegboyega who stressed that family planning the ability of individuals and couples to attain their desired number and spacing of their children through contraceptive use is one of the most cost-effective public health interventions and is pivotal to reducing the country's fertility. Also, Adegboyega established that contraceptive discontinuation was significantly associated with women's educational level.

Finding also revealed that there was no significant difference in the factors influencing use of contraceptives among literate married women in Ogbomoso South Local Government Area, Oyo State based on age. This implies that age has no influence on the contraceptive use among literate married women in Ogbomoso South Local Government Area, Oyo State. This finding agrees with that of Okonofua, who concluded that age did not influence the level of consumption of modern contraceptives as far as people are aware of pregnancy prevention methods.

Another finding revealed that there was no significant difference in the factors influencing use of contraceptives among literate married women in Ogbomoso South Local Government Area, Oyo State based on religion. This could be due to the fact that as far as no religion is against the use of contraceptives. The finding of this study corroborates the finding of Oye-Adeniran, Adewole, Odeyemi, Ekanem and Umoh who revealed that religious affiliation has no influence on the patronage of contraceptives.

Finding revealed that there was a significant difference in the factors influencing use of contraceptives among literate married women in Ogbomoso South Local Government Area, Oyo State based on educational qualification. The finding agrees with Abasiattai, Bassey and Udoma that observations in some centers and communities indicated that staffs in health centers are becoming an important source of information, especially in southern Nigeria. This is probably because of the increased level of education among women and mothers in southern parts of Nigeria. Also, Okpani and Okpani (2000); Oye-Adeniran, Adewole, Odeyemi, Ekanem and Umoh that good knowledge and awareness did not show a strong prevalence of use of contraception.

## Conclusion

Based on the findings of the study, the following conclusions were drawn: The factors influencing contraceptive use among literate married women are educational qualification, health condition and number of children among others. There were no significant differences in the factors influencing use of contraceptives among literate married women in Ogbomoso South Local Government Area, Oyo State based on age and religion while there was a significant difference in the factors influencing use of contraceptives among literate married women in Ogbomoso South Local Government Area, Oyo State based on educational qualification.

## Recommendations

Based on the findings of this study, it is recommended that:

- Contextual and cultural considerations are recommended for comprehensive understanding of factors influencing contraceptive use among Nigerian women.
- Educative interventions by service providers on the necessity of continuous contraception even at older age before menopause should be recommended. In rural settings where accessibility is low, government efforts should be geared towards making contraceptive options available to users in order to allow them make preferred and compatible choices among method(s).
- Government, non-governmental organizations and service providers should subsidize contraceptive methods further in order to spur users who cannot afford the preferred, available or compatible methods.
- Married women should be reoriented, emphasizing the risk of abandonment and health benefits of limited and well-spaced births; hence, the necessity of continuous contraception.
- Programs should be sponsored on radio to target the rural women for information, communication and education focusing on increasing awareness of the benefits of and support for use of contraceptives use.
